# Characterization of Hypomagnesemia in Alcoholic Hepatitis Patients and Its Association with Liver Injury and Severity Markers

**DOI:** 10.3390/jcm12082968

**Published:** 2023-04-19

**Authors:** Evan J. Winrich, Harsh Tiwari, Khushboo S. Gala, Amor J. Royer, Dipendra Parajuli, Vatsalya Vatsalya

**Affiliations:** 1 Department of Medicine, University of Louisville, Louisville, KY 40202, USA; 2 Alcohol Research Center, University of Louisville, Louisville, KY 40202, USA; 3 Clinical Laboratory for Intervention Development of AUD and Organ Severity, University of Louisville, Louisville, KY 40202, USA; 4 Robley Rex Louisville VAMC, Louisville, KY 40206, USA; 5 National Institute on Alcohol Abuse and Alcoholism, Bethesda, MD 20892, USA

**Keywords:** ABIC, alcohol-associated hepatitis, alcohol-associated liver disease, hypomagnesemia, magnesium, MELD, severe AH, mortality

## Abstract

Introduction: Hypomagnesemia has been documented in alcohol-associated liver disease (ALD). This study aims to characterize hypomagnesemia in alcoholic hepatitis (AH) patients and identify its response with liver injury and severity markers. Materials and Methods: A total of 49 male and female AH patients with an age range of 27–66 years were enrolled in this study. Patients were grouped by MELD: MiAH (mild AH < 12 [*n* = 5]), MoAH (12 ≤ moderate AH ≤ 19 [*n* = 13]), and SAH (severe AH ≥ 20 [*n* = 31]). Patients were also evaluated by MELD grouping as non-severe (MELD ≤ 19 [*n* = 18]) and severe (MELD ≥ 20 [*n* = 31]). Data were collected on demographics (Age; BMI), drinking history (AUDIT; LTDH), liver injury (ALT; AST), and liver severity (Maddrey’s DF; MELD; AST:ALT). Serum magnesium (SMg) levels were tested as SOC lab (normal ≥ 0.85 ≤ 1.10 mmol/L). Results: SMg was deficient in each group; the lowest in the MoAH patients. The true positivity of SMg values were at a good performance level when compared between severe and non-severe AH patients (AUROC: 0.695, *p* = 0.034). We found that the SMg level < 0.78 mmol/L could predict severe AH (sensitivity = 0.100 and 1-specificity = 0.000) at this true positivity, and subsequently analyzed patients with SMg < 0.78 mmol/L (Gr.4) and ≥0.78 mmol/L (Gr.5). Between Gr.4 and Gr.5, there were clinically as well as statistically significant differences in disease severity as defined by MELD, Maddrey’s DF, and ABIC scores. Conclusions: This study demonstrates the utility of SMg levels to identify AH patients who may have progressed to severe status. The extent of magnesium response in AH patients also corresponded significantly with the prognosis of liver disease. Physicians suspecting AH in patients with recent heavy drinking may use SMg as an indicator to guide further testing, referrals, or treatment.

## 1. Introduction

Alcohol-associated hepatitis (AH) is an acute, pro-inflammatory dysfunction of the liver associated with heavy alcohol use, which leads to significant morbidity and mortality [1]. A recent consensus statement from the Alcoholic Hepatitis Consortium sponsored by the National Institute of Alcohol Abuse and Alcoholism (NIAAA), provided a working definition of AH as onset of jaundice within 60 days of heavy consumption of alcohol (>50 g/day) for a minimum of 6 months, a serum bilirubin > 3 mg/dL, an elevated AST (50–400 U/L), an AST:ALT ratio > 1.5, and no other obvious cause for hepatitis [2].

Both acute and chronic alcohol intake can lead to a variety of electrolyte imbalances including hypomagnesemia [3]. Hypomagnesemia can occur in patients with alcohol abuse through reduced intestinal absorption, increased urinary losses, and intracellular shift of magnesium [4]. Previous studies have documented hypomagnesemia in early alcoholic liver disease and chronic alcohol abuse [5,6].

However, there remains a paucity of data on electrolyte imbalances in acute alcoholic hepatitis including magnesium. We believe that this is an area of keen clinical interest as our previous studies have shown a reduction in markers of hepatic necrosis and apoptosis after just 2 weeks of standard of care magnesium supplementation in early alcoholic liver disease patients [7]. Thus, understanding the extent of deficiency in the magnesium level in AH is important. Additionally, identifying the level of deficiency during the different stages of AH severity, namely, moderate and severe, could be helpful in strategizing the medical management of this acute pathology. Importantly, we do not know much about the response of magnesium levels when the severity of AH is higher, and the mortality outcomes could have high likelihood under such circumstances.

It has been previously shown that there may be a mortality benefit in patients with liver disease who had a higher intake of magnesium [8]. There are various complications observed in AH patients at its severe stage; the levels of magnesium should be precisely studied to identify how and why the changes in magnesium level will correspond to the staging of ALD including AH (an advanced and acute stage). If complications such as acute renal failure or end stage renal failure are present, the data suggest that magnesium levels, in contrast, may increase [9]. Filling such gaps in the clinical information between serum magnesium level and its relation to liver injury, inflammation, severity, and prognosis in AH patients may provide informed clinical determination.

Our clinical study aims to characterize the deficiency and role of magnesium in acute alcoholic hepatitis during its clinical staging. The second aim of this study was to identify whether there are any prospects of using magnesium levels compared with the present clinical prognostic indicator of AH. We also explored the magnesium response in AH patients who could have low survivability as defined by clinically significant ABIC, a mortality index of severe AH.

## 2. Materials and Methods

This clinical study was a secondary investigation of a single timepoint as an observational paradigm in a tertiary setup for the liver service at the University of Louisville. Study was approved by the local IRB of the University of Louisville #12.0427. All study participants consented under a large national consortia trial, AlcHepNet, before any clinical information and samples were collected. A total of 49 male and female AH patients with an age range of 27–66 years were enrolled in this study. For the initial analysis, patients were grouped based on the MELD (Model for End-Stage Liver Disease) score at project enrollment to approximate the disease severity. Group 1 (Gr.1) consisted of patients with a MELD score < 12 (*n* = 5) and was termed as mild alcohol hepatitis (miAH). Group 2 (Gr.2) consisted of patients with MELD scores ≥ 12 and ≤19 (*n* = 13) and was termed moderate alcohol hepatitis (moAH). Group 3 (Gr.3) consisted of patients with a MELD score ≥ 20 (*n* = 31) and was termed severe alcohol hepatitis (SAH). Patients were also evaluated by MELD grouping as non-severe (MELD ≤ 19 [*n* = 18], nSAH) and severe (MELD ≥ 20 [*n* = 31], SAH). Comprehensive details on the eligibility and exclusion criteria are primarily available in the NCT registry and previous publications [10,11,12,13].

Data were collected on demographics (age in years, and BMI), drinking history (AUDIT [14] and LTDH [years of drinking] [15]), liver injury (alanine transaminase, ALT; aspartate transaminase, AST), and liver severity (Maddrey’s discriminant function [16], MELD score [17], ABIC (Age, Bilirubin, INR, Creatinine) score [18]), and progression (AST:ALT). Response to standard of care steroid treatment after seven days was also collected via the Lillie score [19]. Serum magnesium (SMg) levels were tested as SOC lab (normal ≥ 0.85 ≤ 1.10 mmol/L). None of the participants in this study received prior or ongoing SOC magnesium at the time of consenting and sample collection.

Between-group factorial ANOVA, regression linear models (univariate, multivariable) and AUROC statistical models were used in this study. Data assembly and processing were performed using Microsoft Excel (MS 365, Microsoft Corp., Redmond, WA, USA), and data analyses were performed using SPSS 28.0 version (IBM, Armonk, NY, USA). Statistical significance was set at *p* < 0.05. Data were presented as the mean ± standard deviation unless specifically mentioned otherwise.

## 3. Results

### 3.1. Analysis of Liver Injury and Severity Based on Disease Severity

Evaluation of the demographic data, drinking history, liver injury, liver disease severity, and response to standard of care steroid treatment based on AH disease severity (Gr.1, Gr.2, Gr.3) was performed as seen in Table 1.

#### 3.1.1. Demographics and Drinking History

There was no statistically significant difference in the age or BMI between groups at the baseline. The Alcohol Use Disorders Identification Test (AUDIT) scores were obtained on each patient upon entry of the study to quantify recent drinking history within the last year, and lifetime drinking history (LTDH) was recorded to assess the total years of heavy drinking. There was no significant difference in the AUDIT score or LDTH between the three groups. Thus, the participants were very similar in their alcohol intake profile both in terms of their lifetime and past year alcohol assessments.

#### 3.1.2. Liver Injury Markers and Serum Magnesium Levels

**Figure 1:**
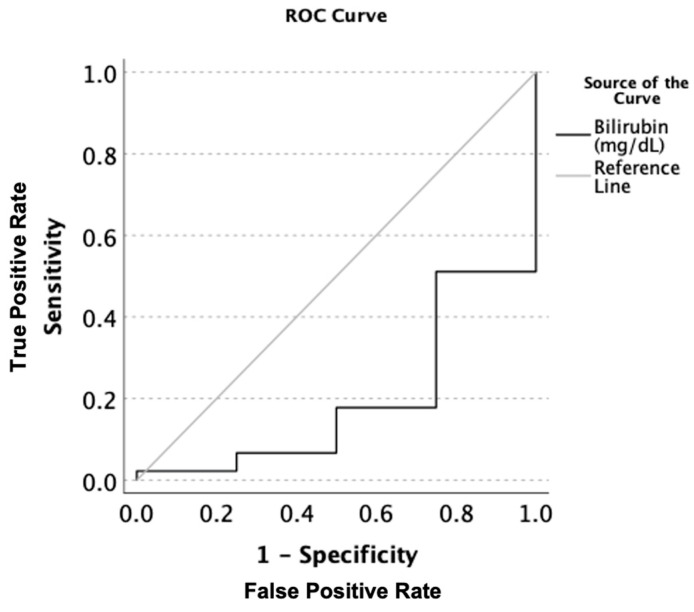
AUROC analysis of the serum total bilirubin levels (mg/dL) in all patients with alcoholic hepatitis when comparing magnesium deficient to magnesium non-deficient patients. Sensitivity describes the ability of this test to correctly identify patients with hypomagnesemia using total serum bilirubin. 1-Specificity is the ability of this test to correctly identify participants in this study without hypomagnesemia in the context of the total serum bilirubin.

**Figure 2:**
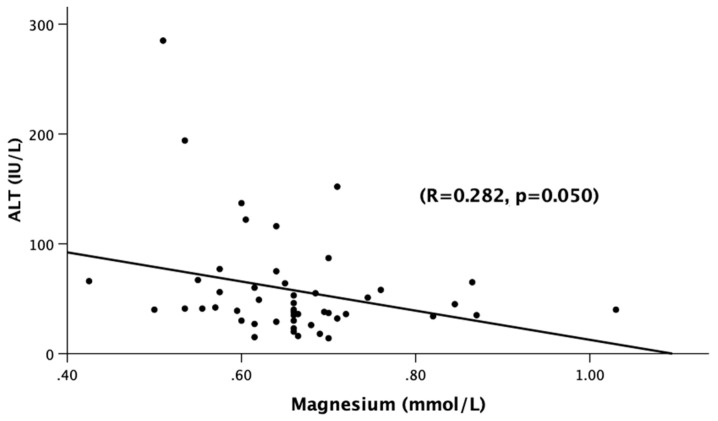
Association of serum magnesium levels (mmol/L) and ALT (IU/L) among all of the alcoholic hepatitis patients (Gr.1, Gr.2, Gr.3). Statistical significance was set at *p* < 0.05. The correlational coefficient (R) is provided.

There was a statistically significant difference in the total bilirubin (Tbili) levels across the three groups. TBili corresponded with a very good stage of significant true positivity in all of the AH patients when compared to the normal or low Mg (AUROC: 0.806, *p* = 0.003 [CI: 0.602–1.009], Tbili: 3.1 [sensitivity = 1.0, and 1-specificity = 0.844]) (Figure 1). Assessment of the liver panel revealed no significant difference in ALT, AST, or AST:ALT between any of the groups, suggesting that the patients’ ongoing injury was more-or-less similar. Notably, there was a significant inverse correlation between the serum magnesium and ALT (Figure 2), suggesting the role of deficient magnesium with the liver injury marker, ALT.

#### 3.1.3. Liver Disease Severity

**Figure 3:**
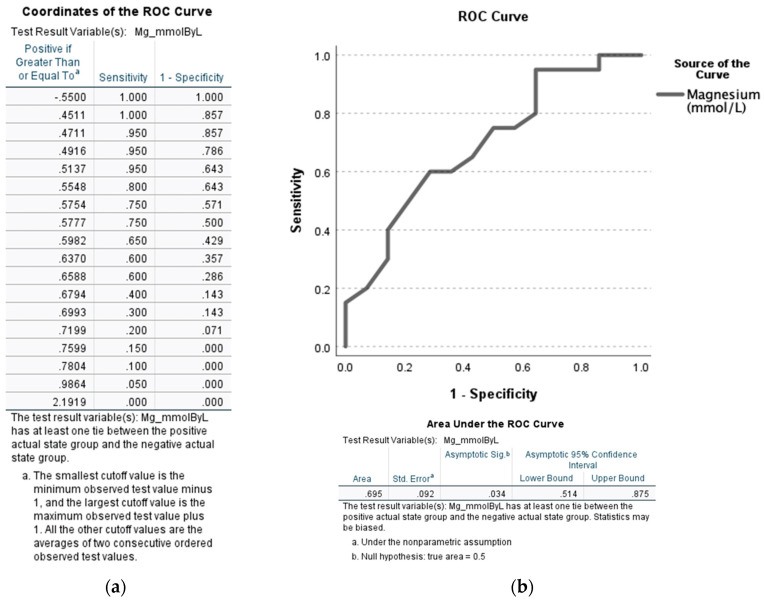
AUROC analysis of the serum magnesium levels (SMg [mmol/L]) among severe alcoholic hepatitis patients (MELD > 19) when compared to non-severe alcoholic hepatitis (MELD ≤ 19). (**a**) Coordinates of the ROC curve. (**b**) ROC curve with confidence interval (lower and upper bound) and area under the curve (area) provided at the bottom of the figure. Statistical significance was set at *p* < 0.05. Sensitivity describes the ability of this test to correctly identify severe AH patients with hypomagnesemia. 1-Specificity is the ability of this test to correctly identify the severe AH participants in this study with hypomagnesemia in the context of MELD scores.

As seen in Figure 3, the true positivity of SMg values were at a good performance level when compared between severe and non-severe AH patients (AUROC: 0.695, *p* = 0.034 at SMg: 0.7804 [sensitivity = 0.100 and 1-specificity = 0.000]). Given the ability of serum magnesium to predict severe alcoholic hepatitis at a level of <0.78 mmol/L, we decided to further analyze the characteristics of patients both above and below this serum level.

### 3.2. Analysis of Liver Injury and Severity Based on Specificity and Sensitivity of Serum Magnesium

For the subsequent analysis, all of the participating AH patients were re-evaluated by grouping them based on the serum magnesium level cutoff derived from the AUROC results in Figure 3. The justification for this testing was focused on whether the patients with AH could be detected with confidence for their disease when the hypomagnesemia was below 0.78 mmol/L. Group 4 (Gr.4) consisted of patients with serum magnesium ≥ 0.78 mmol/L (*n* = 5) and Group 5 (Gr.5) consisted of patients with serum magnesium < 0.78 mmol/L (*n* = 44) by using this criteria. This was performed by picking a representation of a sensitivity/specificity pair corresponding (and immediate higher, SMg = 0.7804) to a particular decision threshold (which was SMg = 0.7599 when the false positive rate was 0.000 for 1-specificity). The same evaluation of demographic data, drinking history, liver injury, liver disease severity, and response to standard of care steroid treatment was performed, as seen in Table 2.

#### 3.2.1. Demographics and Drinking History

There was no statistically significant difference in the age or BMI characteristics between Gr.4 and Gr.5, indicating a homogenous baseline cohort. Additionally, each group had similar long-term drinking history. There was no significant difference in the AUDIT score or LDTH between the three groups. The evaluation of between sex differences was limited by a lack of female patients in Gr.4.

#### 3.2.2. Liver Injury Markers

**Figure 4:**
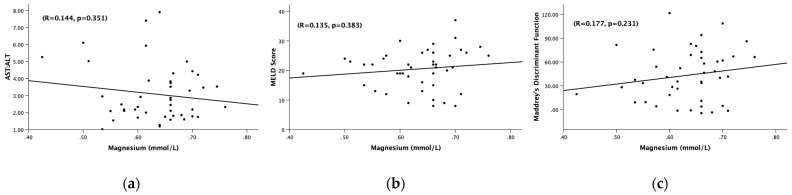
Association of the serum magnesium levels (mmol/L) and (**a**) AST:ALT ratio, (**b**) MELD score, (**c**) Maddrey’s discriminant function. (**a**) Association of the serum magnesium levels (mmol/L) and AST:ALT ratio among hepatitis patients with serum Mg < 0.78 mmol/L. (**b**) Association of the serum magnesium levels (mmol/L) and MELD score among all alcoholic hepatitis patients with serum Mg < 0.78 mmol/L. (**c**) Association of serum magnesium levels (mmol/L) and Maddrey’s discriminant function among all alcoholic hepatitis patients with serum Mg < 0.78 mmol/L. Statistical significance was set at *p* < 0.05. Correlational coefficient (R) is provided.

There was a clinically notable and statistically significant difference in the total bilirubin between Gr.4 and Gr.5 (*p* = 0.001). Gr.4 had a mean total bilirubin of 22.58 ± 10.38 mg/dL, while Gr.5 had a mean total bilirubin of 10.28 ± 7.23 mg/dL (Table 2). There was not a significant difference in ALT, AST, or AST:ALT between Gr.4 or Gr.5. We did not find any significant association of serum magnesium with the AST:ALT ratio, MELD, and Maddrey’s DF in Gr.5 (Figure 4). It is likely that the magnesium cutoff is primarily effective in indicating the presence of AH more than detailing the characterization of the clinical markers within the cohort.

#### 3.2.3. Liver Disease Severity

Further notable outcomes were found upon analyzing the liver severity scores when grouping patients by the serum magnesium levels. There were statistically significant differences in the MELD, Maddrey’s DF, and ABIC scores between Gr.4 and Gr.5, as seen in Table 2. Notably, patients in Gr.4 had significantly higher liver severity scores and a poorer prognosis. Gr. 4 showed more than 75% mortality indication by ABIC score and had high MELD, but had magnesium levels that showed an unanticipated paradoxical increase.

**Figure 5:**
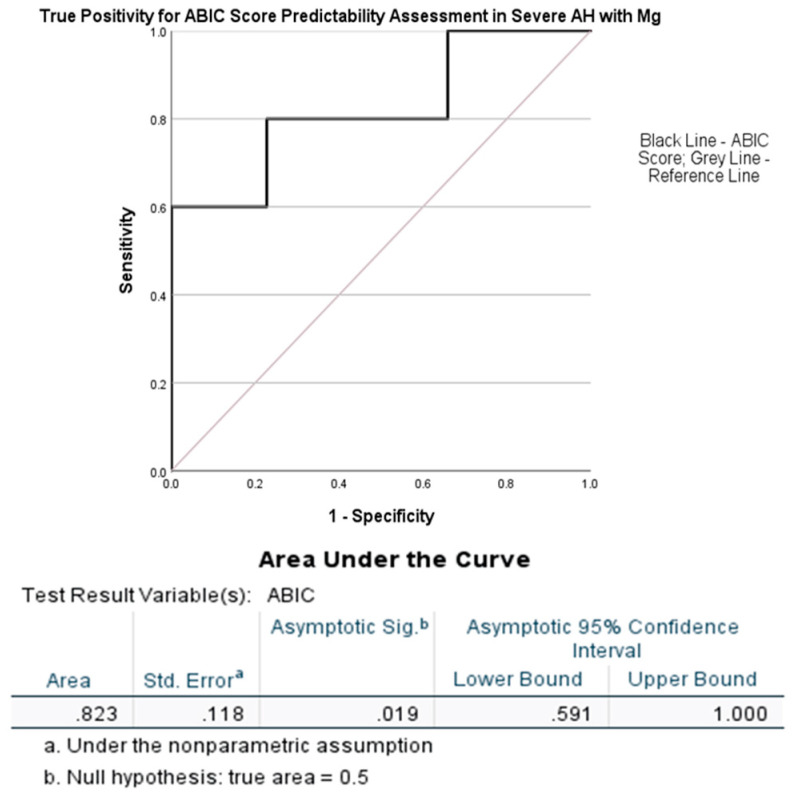
AUROC analysis of ABIC score by the serum magnesium levels (SMg [mmol/L]) at a cutoff of 0.78 mmol/L. Statistical significance was set at *p* < 0.05.

A higher ABIC score (more than 75% mortality within 1 month) that predicts low survivability could be shown at a greater true positive level fit of 0.823 at *p* = 0.019 with a magnesium cutoff chosen at 0.78 mmol/L (Figure 5). All five AH patients with the magnesium levels of Gr. 4 were also very severe in the entire cohort of severe AH patients from the initial analysis (Table 1).

## 4. Discussion

Magnesium is an important micronutrient in our body that plays a role in cellular energy metabolism, DNA transcription, protein synthesis, and electrolyte balance [20]. Factors contributing to hypomagnesemia in patients with acute and chronic alcohol abuse include a poor nutritional status leading to decreased absorption [21,22,23], increased urinary losses [22,23,24], and impaired magnesium homeostasis [25]. Our previous study noted an independent association of lower magnesium levels with higher levels of hepatic apoptosis (quantified by K18M30) and necrosis (quantified by K18M65) in early alcoholic liver disease patients admitted for an inpatient rehabilitation program [7]. Considering that the magnesium levels corresponded with increased liver cell death in these patients, it also led to our investigation of magnesium as a possible prognostic indicator in acute alcoholic patients.

On our initial analysis, we confirmed that the MiAH, MoAH, and SAH groups had homogenous demographic characteristics including age, BMI, and alcohol drinking profile, both in their lifetime (LDTH) and past year (AUDIT) alcohol assessments. Thus, these patients have a very similar presentation for the demographics and drinking profile during the intake assessments. In this situation, it is more challenging to differentially diagnose patients with AH that has a uniquely characterized liver pathology, with rapid onset and progression. However, there was a notable inverse correlation between the serum magnesium levels (which was deficient) and markers of liver injury including ALT (as seen in Figure 2), which was elevated in almost all AH patients, revealing a wider complex pathology than what is presented with liver injury. Thereafter, we evaluated whether a relationship existed between the extent of hypomagnesemia and the liver severity scores including MELD. Perhaps most importantly, we found that the serum magnesium levels could accurately predict those with alcoholic hepatitis in general at a serum magnesium level of <0.78 mmol/L when the ABIC scores are not critical.

Earlier, we reported that such levels of magnesium (sub-clinical) are also present in AUD patients with early-stage ALD or no liver injury [7], thus such levels may not conclude differentiating AUD patients who may have AH or not in an initial clinical review of laboratory charts. We know that when the severity of alcohol-associated liver disease is higher, the homeostasis of magnesium is altered [26]. In such conditions, serum magnesium could be relatively higher due to heavy damage to its transport mechanism, which is primarily regulated by the liver as well as due to the fall in the local magnesium within acute inflamed tissues [27] including the lowering of magnesium within inflamed hepatocytes [28] and intracellular shifts in general [29]. An outward shift of magnesium from intracellular concentrations to serum (or less absorption) due to a lack of transport [30], pro-inflammatory status [31], and disruption of energy mechanisms [32] could be possible reasons for such elevation in very severe AH patients. Indeed, a severity outlier effect that is randomly observed in clinic for the magnesium level when it is higher in very severe AH patients (MELD more than 30) is just not a clinical artifact, but is also indicative of poor prognosis. Importantly, this indicates the utility of the serum magnesium levels to identify patients with AH in clinical scenarios, where liver function testing is not readily available or had not been drawn. Outpatient physicians with a suspicion of AH in patients with recent heavy drinking may use serum magnesium as an indicator to order these liver function and severity associated tests to confirm AH, to admit patients for AH treatment, or provide appropriate treatment referral to a tertiary care setting. Serum magnesium levels could not only be used as an adjunct diagnostic tool in AH, but also as a simple prognostic tool under such pathological staging of the liver.

Circulating Mg represents only about 1% of the body Mg content. Hence, although ionized magnesium is a superior method of measuring the total body magnesium, studies have shown that serum magnesium is still a valuable indicator for clinically significant magnesium levels [33]. It is important to note that subtle hypomagnesemia may be missed by using serum Mg measurements. Newer research has been looking at different stages/levels of magnesium deficiency and has found that even subclinical magnesium deficiency may be one of the leading causes of chronic diseases and early mortality [34]. Clinical implications of hypomagnesemia in liver disease are significant and may lead to supplementation being one of the recommended modalities in the medical management of alcoholic hepatitis.

Nonetheless, in circumstances when the ABIC scores are critically high, we found the completely opposite response of magnesium when it was borderline low to normal. Indeed, the algorithm validates that higher magnesium levels lose the traction to predict AH. In this case, we only had severe AH patients who happened to have borderline low to normal magnesium levels. Thus, in our study, we found both the dimensions of magnesium levels found in AH. With the diagnosis of AH, hypomagnesemia is clearly the outcome of our primary aim. On the other hand, when the severity of AH is very high and the ABIC scores are clinically significant to support negative prognosis, then the magnesium levels actually hovered above 0.78 mmol/L. This could be attributed to the shunting of magnesium transport due to the severity of liver, which may have complications or acute renal function (one of the four aspects of ABIC when clinically significant). The inhibition of local absorption in tissue may occur, resulting in pseudo-elevation of the serum levels. This was a finding reported more than 60 years ago [35] and still presents as a unique presentation in AH patients with higher MELD and ABIC. This was a significant finding in our study cohort, when the MELD and ABIC is too high, then the predictability of magnesium is lost. As generally anticipated to be hypomagnesemia exhibited in otherwise AH patients, we found a total inverse shift in the magnesium response [36].

Studies in alcohol fed rats have shown that magnesium supplementation helped with oxidative stress and tissue damage, as evidenced by the elevated total antioxidant status (TAS) in serum, activity of glutathione peroxidase, and the ratio of reduced glutathione to oxidized glutathione (GSH/GSSG) in the liver as well as tissue histopathological changes [37]. Magnesium supplementation in rats with liver damage has been shown to have anti-fibrotic properties, with an improvement in the oxidant and antioxidant parameters and histopathological examination [38]. One trial of magnesium supplementation on human chronic alcohol users in Finland showed that when given magnesium supplementation, patients had lowered serum AST levels and may have a decreased risk of death from alcoholic liver disease [39]. In fact, one study showed that compared to patients with non-alcoholic steatosis, patients with steatohepatitis had lowered serum magnesium levels [40]. Findings from this study could pave the way for the consideration of magnesium supplementation as an adjunct treatment with a further understanding of this disease state that has few treatment options in current clinical practice.

There were several limitations in this study. The sample size of this clinical study resembled that of a pilot study cohort. Thus, detailed outcomes from this study were not within the scope. This was also not a longitudinal study; thus, the treatment course of SOC or the natural history of AH patients was not collected. In the second analyses, the availability of female participants limited the scope of outcomes listed in Table 2. We also only had five patients in Gr.4 who had borderline to normal serum magnesium levels. Such a low number limited the scope of any further extensive analysis other than the group differences or true positivity indices. We also did not test for any biomarkers, though this is a keen interest of the authors and could be a future study direction.

Previously, we found that hypomagnesemia in the early stages of alcohol-associated liver disease could be one of the factors leading to progression of the disease [3]. In our subsequent clinical longitudinal study on early-stage ALD, we found that magnesium levels could be a valuable indicator of liver cell death and the recovery of liver health with standard of care magnesium supplementation in AUD patients with early-stage liver disease [7]. In this study, we found that there could be a deficiency level of magnesium that would provide vital clues for the detection of AH. Importantly, a paradox increase in magnesium level could be observed in AH patients with high MELD and high ABIC. These studies warrant further treatment trials and large longitudinal studies to explore the potential of magnesium as a frontline therapeutic or adjunct therapy of liver disease including AH.

## Figures and Tables

**Table 1:** Demographic, drinking, liver injury and severity markers in AH patients grouped by the categorical ranking of MELD score. Statistical significance was set at *p* < 0.05. Significant measures are in bold. Data are presented as the mean ± standard deviation.

Measures	Group 1 (Mild AH; MELD < 12)			Group 2 (Moderate AH; MELD ≥ 12 and ≤19)			Group 3 (Severe AH; MELD > 19)			Between Group *p*-Value
	Males (*n* = 3; 6.12%)	Females (*n* = 2; 4.08%)	Total (*n* = 5; 10.20%)	Males (*n* = 9; 18.37%)	Females (*n* = 4; 8.16%)	Total (*n* = 13; 26.53%)	Males (*n* = 21; 42.86%)	Females (*n* = 10; 20.41%)	Total (*n* = 31; 63.27%)	
Demographic Data										
Age (years)	59.0 ± 2.2	52.5 ± 13.5	56.40 ± 9.27	49.4 ± 6.9	48.3 ± 7.1	49.1 ± 7.0	47.5 ± 10.8	42.3 ± 8.8	45.8 ± 10.5	0.083
BMI (kg/m^2^)	29.8 ± 6.2	27.1 ± 5.1	28.7 ± 5.9	30.2 ± 7.7	27.9 ± 5.7	29.5 ± 7.2	29.6 ± 6.13	25.3 ± 6.8	28.2 ± 6.6	0.854
Drinking History										
AUDIT	22.7 ± 13.0	15.5 ± 2.5	19.8 ± 10.8	22.4 ± 7.1	20.3 ± 8.6	21.8 ± 7.6	21.9 ± 8.0	23.9 ± 6.8	22.5 ± 7.7	0.787
**LTDH** ^a^	41.3 ± 3.1	32.5 ± 11.5	37.8 ± 8.8	25.1 ± 7.5	16.3 ± 9.0	22.9 ± 8.8	22.8 ± 12.3	25.3 ± 9.0	23.6 ± 11.4	**0.028**
Serum Magnesium Level										
Magnesium (mmol/L)	0.65 ± 0.033	0.68 ± 0.028	0.66 ± 0.032	0.59 ± 0.080	0.63 ± 0.033	0.60 ± 0.071	0.70 ± 0.129	0.65 ± 0.067	0.68 ± 0.115	0.056
Markers of Liver Injury										
**Bilirubin (mg/dL)**	1.40 ± 0.26	2.00 ± 0.57	1.64 ± 0.47	7.51 ± 5.50	4.43 ± 0.85	6.56 ± 4.75	16.47 ± 8.07	12.57 ± 7.06	15.21 ± 7.86	**<0.001**
ALT (IU/L)	46.3 ± 14.7	38.5 ± 1.5	43.2 ± 12.0	108.9 ± 45.6	28.8 ± 10.2	84.2 ± 53.3	53.0 ± 53.2	40.9 ± 22.5	49.1 ± 46.0	0.655
AST (IU/L)	84.0 ± 29.4	88.0 ± 22.0	85.6 ± 26.8	206.6 ± 97.8	104.8 ± 24.4	175.2 ± 94.4	198.8 ± 278.3	113.1 ± 57.3	171.2 ± 234.7	0.072
AST:ALT	1.81 ± 0.17	2.27 ± 0.46	1.99 ± 0.40	2.08 ± 1.25	4.21 ± 1.94	2.74 ± 1.79	3.59 ± 1.41	3.25 ± 1.50	3.48 ± 1.44	0.083
Liver Severity Scores										
MELD	8.7 ± 0.5	9.0 ± 1.0	8.8 ± 0.75	15.3 ± 2.9	17.8 ± 1.6	16.1 ± 2.8	26.9 ± 5.7	25.1 ± 5.0	26.3 ± 5.51	N/A
**Maddrey’s DF**	−3.0 ± 1.3	4.3 ± 0.4	−0.1 ± 3.7	18.9 ± 24.5	28.4 ± 11.3	21.8 ± 21.8	74.5 ± 50.5	63.7 ± 19.8	71.0 ± 43.4	**<0.001**
**Child Pugh Score**	5.6 ± 0.5	6.5 ± 1.5	6.0 ± 1.1	8.9 ± 1.5	9.5 ± 0.9	9.1 ± 1.4	10.9 ± 1.1	11.1 ± 1.4	11.0 ± 1.2	**<0.001**
**ABIC Score**	7.13 ± 0.23	6.52 ± 2.01	6.89 ± 1.07	6.77 ± 0.79	6.71 ± 0.93	6.75 ± 0.79	8.27 ± 1.43	7.25 ± 1.14	7.94 ± 1.41	**0.012**
Lille Score ^b^	0.93 ± N/A	N/A ± N/A	0.93 ± N/A	0.80 ± 0.20	0.83 ± 0.08	0.81 ± 0.17	0.89 ± 0.20	0.72 ± 0.29	0.83 ± 0.24	0.890

^a^ LTDH: total *n* = 46; ^b^ Lille score: total *n* = 26; group 1 males *n* = 1; group 1 females *n* = 0. AH: alcoholic hepatitis; BMI: body mass index; AUDIT: Alcohol Use Disorders Identification Test; LTDH: sum of total drinking years; N/A: not applicable.

**Table 2:** Demographic, drinking, liver injury and severity markers in AH patients grouped by magnesium level at a cutoff of 0.078 mmol/L. Statistical significance was set at *p* < 0.05. Significant measures are in bold. Data are presented as the mean ± standard deviation.

Measures	Group 4 (Patients with Mg ≥ 0.78 mmol/L)			Group 5 (Patients with Mg < 0.78 mmol/L)			Between Group *p*-Value
	Males (*n* = 5; 10.20%)	Females (*n* = 0; 0%)	Total (*n* = 5; 10.20%)	Males (*n* = 28; 57.14%)	Females (*n* = 16; 32.65%)	Total (*n* = 44; 89.80%)	
Demographic Data							
Age (years)	44 ± 4	N/A	44 ± 4	50 ± 11	45 ± 10	48 ± 11	0.339
BMI (kg/m^2^)	29.84 ± 8.50	N/A	29.84 ± 8.50	29.77 ± 6.52	26.17 ± 6.62	28.46 ± 6.71	0.672
Drinking History							
AUDIT	23.30 ± 10.47	N/A	23.30 ± 10.47	21.89 ± 8.31	21.94 ± 7.71	21.91 ± 8.00	0.741
LTDH ^a^	20.80 ± 8.50	N/A	20.80 ± 8.50	26.04 ± 12.47	24.43 ± 11.00	25.59 ± 11.87	0.399
Serum Magnesium Level							
Magnesium (mmol/L)	0.89 ± 0.083	N/A	0.89 ± 0.083	0.63 ± 0.075	0.65 ± 0.057	0.63 ± 0.069	N/A
Markers of Liver Injury							
**Bilirubin (mg/dL)**	22.58 ± 10.38	N/A	22.58 ± 10.38	10.88 ± 7.35	9.21 ± 7.11	10.28 ± 7.23	**0.001**
ALT (IU/L)	43.80 ± 12.64	N/A	43.80 ± 12.64	71.86 ± 59.65	37.56 ± 19.81	59.39 ± 51.47	0.507
AST (IU/L)	150.80 ± 35.74	N/A	150.80 ± 35.74	197.57 ± 253.72	107.87 ± 49.83	164.95 ± 207.83	0.881
AST:ALT	3.55 ± 0.90	N/A	3.55 ± 0.90	2.92 ± 1.61	3.37 ± 1.70	3.08 ± 1.64	0.532
Liver Severity Scores							
**MELD**	33.40 ± 8.26	N/A	33.40 ± 8.26	20.07 ± 6.55	21.25 ± 7.09	20.50 ± 6.70	**<0.001**
**Maddrey’s DF**	116.19 ± 89.66	N/A	116.19 ± 89.66	40.84 ± 34.62	47.45 ± 28.57	43.24 ± 32.37	**<0.001**
Child Pugh Score	10.80 ± 1.10	N/A	10.80 ± 1.10	9.71 ± 2.11	10.13 ± 2.09	9.86 ± 2.09	0.331
**ABIC Score**	9.27 ± 1.76	N/A	9.27 ± 1.76	7.49 ± 1.15	7.02 ± 1.15	7.32 ± 1.15	**0.001**
Lille Score ^b^	0.93 ± 0.10	N/A	0.93 ± 0.10	0.85 ± 0.21	0.74 ± 0.26	0.81 ± 0.23	0.398

^a^ LTDH: total *n* = 46; ^b^ Lille score: total *n* = 26. BMI: body mass index; AUDIT: Alcohol Use Disorders Identification Test; LTDH: sum of total drinking years; N/A: not applicable.

## Data Availability

Data can be provided by contacting the corresponding author with the appropriate requests.

## Abbreviations

AH	Alcohol-associated hepatitis
AUD	Alcohol use disorder
AUDIT	Alcohol use disorders identification test
CS	Clinically significant
LTDH	Lifetime drinking history
NCS	Clinically non-significant
ABIC	Algorithm made from age, serum bilirubin, INR, and serum creatinine
serum ALT	Alanine aminotransferase
serum AST	Aspartate aminotransferase
AUROC	Area under the receiver operating characteristics
SM	Serum magnesium
SOC	Standard of care
